# Introduction to the special issue in honor of Luigi Orsenigo

**DOI:** 10.1007/s00191-021-00751-6

**Published:** 2021-11-18

**Authors:** Franco Malerba, Uwe Cantner, Stefano Breschi

**Affiliations:** 1 Department of Management and Technology, Bocconi University, Milan, Italy; 2 Department of Economics, Friedrich Schiller University Jena, Jena, Germany; 3 Department of Marketing and Management, I2M Group, University of Southern Denmark, Odense, Denmark

Luigi Orsenigo passed away in May 2018, leaving all of us with a major loss. This special issue in his honour brings several contributions in the areas in which Gigi contributed in a significant way in the course of his career: the pharmaceutical and biotechnology industries, innovation and industrial dynamics, evolutionary theory and modelling.

After graduating in Economics at Bocconi University, Luigi obtained a Ph.D. from the Science Policy Research Unit (SPRU), University of Sussex. Over the course of his career, he has been affiliated with Bocconi University, the University of Brescia and the Open University. At the moment of his death, Luigi Orsenigo was R.M. Phillips Professor of Economics of Innovation at SPRU and Professor of Applied Economics at IUSS (Institute for Advanced Studies) at Pavia. He had also been Fellow of Cespri first, and ICRIOS later, both at Bocconi University. For a long time, he has served as Editor of the Journal of Evolutionary Economics, Advisory Editor of Research Policy and Associate Editor of Industrial and Corporate Change, the main journals devoted to economics of innovation and industrial change. He also has been advising several Italian and international institutions on matters of innovation and industrial policy, including the European Commission and the World Intellectual Property Organization (WIPO).

Luigi has been the author of four books and has published many articles on major international journals. The bibliography at the end of this introduction includes his main contributions. In 2012, he has been awarded the Schumpeter Prize for the book “Innovation and the Evolution of Industries. History Friendly Models”, together with F. Malerba, R. Nelson and S. Winter, Cambridge University Press.

Since his graduation from SPRU Luigi has consistently developed a series of lines of research that have span over a set of areas that are broad but interconnected. One of us, Franco Malerba, *in this Special Issue* reconstructs Luigi’s intellectual development and also provides personal memories.

In this Special Issue we want to honour Luigi’s memory with contributions that are related to his research. The papers are written by some of the key co-authors of Orsenigo and reflect his main areas of research.

A first area of major research by Luigi Orsenigo, a real *file rouge* during his life, concerns innovation and the evolution of the pharmaceutical and biotechnology industries, with the role of science playing a prominent role. Among the many papers and book written by Luigi on this topic, here we want to mention *“The Emergence of Biotechnology. Institutions and Markets in Industrial Innovation”*, Pinter Publishers, London, 1989; “Technological Change and the Dynamics of Networks of Collaborative Relations. The Case of the Bio-pharmaceutical Industry”, (with F. Pammolli and M. Riccaboni), *Research Policy*, 2001; *“The Economics of Biotechnology*”, (with M. McKelvey, ed.), Edward Elgar, Cheltenham, 2006.

Luigi has been interested in the biotechnology revolution and the changes that have consequently occurred in the pharmaceutical industry. Due to this revolution, an industry dominated by a stable core of large firms moved to a market structure in which a division of scientific, innovative and production labour has taken place between large incumbent firms and innovative start-ups. This major change and the evolution that followed is also related to a new role that science has taken place in the pharmaceutical industry and to the new relevance of universities and university spin-offs. This new scientific knowledge base changed the type of competition among large incumbents and entrants and led to the rise of networks of collaborations in R&D among actors of different types. Luigi also examined the changes in the industry in the most recent period, in which some of the new entrants have become also integrated pharmaceutical companies, while at the same time some of the large pharmaceutical companies developed their own in-house capabilities in science and biotechnology. In all these dynamics, the high appropriability conditions that characterize the pharmaceutical industry, in which patents play a major role, have fostered and also limited technological change, competition and economic development, in various and complex ways.

Two papers in this Special Issue focus on the pharmaceutical industry and on the role of scientists and R&D and knowledge networks. Bastian Rake, Pablo D’Este and Maureen McKelvey in *Exploring Network Dynamics in Complex Fields of Science: The Formation of Ties to Knowledge Translators* **s**tart from Luigi’s interest in the analysis of the relationship between individual scientists, knowledge evolution and the structure of the pharmaceutical industry. In a sample of 9543 cancer clinical trials over the period 2002–2012 they examine how changes in networks are influenced by the structure of networks as well as the behaviours and characteristics of key individual scientists. Using temporal exponential random graph models, they examine whether the mechanisms of preferential attachment, multi-connectivity, and homophily drive the formation of new collaborative relations to investigators who have knowledge in basic and clinical research. Results indicate that, first, the fragmentation of the network remains high, due to a considerably increasing number of investigators in the network and, secondly, that this fragmentation limits opportunities for knowledge transfer. Homophily in research fields and country of investigators’ affiliation as well as heterophily in terms of publication output are drivers for tie formation to these knowledge translators.

Fabio Pammolli, Massimo Riccaboni and Alessandro Spelta in *The Network Origins of Schumpeterian Innovation* investigate the evolving division of innovative labour in biotechnology and pharmaceuticals from 1981 to 2012, with particular reference to the roles of large and small firms. Relying on topological methods they find that, while a regime of polarization through preferential attachment driven by large pharmaceutical companies dominated the early stages of the biotechnology revolution, in recent years the evolution of the collaborative network has been shaped by roles’ transitions between originators and developers of innovative ideas. In particular, starting from the early 2000s, the emergence of general purpose research technologies and the scientific and technological transformations in genomics have led to a promiscuity of roles, as small and large firms act both as originators and developers, in a less polarized network. The authors then propose a parsimonious model of network formation and evolution able to account for some of the features of the processes underlying the evolution of the network.

A second area of Orsenigo’s research regards the factors affecting the diversity in the patters of innovative activities across industries and the role of the learning environment in affecting these patterns. In several of his work, Orsenigo used the concept of technological regime as one key factor affecting market structure in an industry. Among his various contributions on the issue, one could mention “Schumpeterian patterns of innovation”, (with F. Malerba), in *Cambridge Journal of Economics,* 1995; “Technological Regimes and Sectoral Patterns of Innovative Activities” (with F. Malerba), *Industrial and Corporate Change,1997* and “Technological regimes and Schumpeterian patterns of innovation” (with S. Breschi and F. Malerba), *The Economic Journal*, April, 2000. In these and other contributions the view that the specific properties of the technology of an industry in terms of opportunity, appropriability and cumulativeness conditions and of the knowledge base affect the industrial patterns of innovation is proposed. In particular, a technological regime in which opportunity conditions are high, the appropriability of innovation is low and the cumulativeness of innovation lead to a sectoral pattern supportive to new firms, turbulent industrial dynamics and a changing hierarchy of major innovators (Schumpeter Mark I); on the contrary, a technological regime characterized by medium or high technological opportunities, high appropriability and cumulativeness are conducive to a more stable and concentrated industrial pattern of innovation (Schumpeter Mark II). These relationships have been discussed at the theoretical level and in several contributions tested empirically for many technologies, industries and countries.

In this special issue, Roberto Fontana, Arianna Martinelli and Alessandro Nuvolari in *Regimes reloaded! A reappraisal of Schumpeterian patterns of innovation, 1977–2011* revisit this concept and perform a quasi-replication of the original empirical exercise. By using more recent data and an expanded dataset of innovations in several industries and countries compared to the original ones of the 1990s and early 2000, they confirm that that the distinction between Schumpeterian Mark I and Mark II patterns of innovation and their explanation in terms of technological regimes has still a major validity and yields relevant insights concerning on the connection between inventive activities and industrial dynamics.

A third area of inquiry has concerned industrial dynamics, and particular firms’ persistence in innovative activity and the entry and exit of innovators. Among Luigi’s papers, we would like to highlight “The Dynamics and Evolution of Industries” (with F. Malerba), *Industrial and Corporate Change*, Vol.5, n.1, 1996; “Industrial Dynamics: Stylized Facts, Empirical Evidence and Theoretical Interpretations” (with G. Dosi and F.Malerba), *Industrial and Corporate Change, 1997,* “Technological Entry, Exit and Survival” (with F. Malerba), *Research Policy*, 1999; “The Persistence of Innovative Activities: A Cross-Country and Cross-Sectors Comparative Analysis” (with E. Cefis), *Research Policy*, 2001. Industrial dynamics has been identified by Luigi as one of the fundamental aspects of change in the economies and the typical example of Schumpeterian competition. In his empirical work, Luigi has examined the processes that lead to the entry and exit of innovators across a broad range of technologies and the persistence of those firms that continue of innovate over time. Moreover, the analysis has been conducted at the micro level over a long period of time and has used patent data at the firm level across different technologies and different countries. The main findings are that the entry and exit processes are technology specific and that a lot of persistency of innovative activities is usually present. However persistency is not very high in the aggregate and declines as time goes by.

Along these lines, in this Special Issue Elena Cefis, Franco Malerba and Orietta Marsili present a paper initially discussed with Luigi, “Revolving door effect” or “Schumpeterian gale of creative destruction?”. The authors claim that two basic patterns of exit can be identified from the literature: the revolving door and the gale of creative destruction. In the first, the liability of newness is a driver of the exit process, while in the second it is the displacement of non-innovators. The authors test these two patterns of exit on the population of Dutch firms exiting in 2018. They find confirmation that exit is industry specific: in fact, these two patterns characterize different types of industries. In industries in which innovation does not play a major role, the revolving door effect is the typical pattern and exit is concentrated among the adolescent and small firms. On the contrary, in industries in which innovation is relevant, exit takes place both among infant as well as mature firms and does not necessarily involve only the smaller firms. In particular, in a highly innovative environment, the exit of mature firms is driven by the innovation of young firms as a case of gale of creative destruction.

As an evolutionary economist, Luigi has often emphasized the widespread and persistent heterogeneity of firms in innovation and diffusion. On this theme, Luigi published “Innovation, Diversity and Diffusion: A Self-Organisation Model”, (with G. Silverberg and G. Dosi), *The Economic Journal*, 1988, a model with firms heterogeneous in their behaviour and with feedback loops driving the diffusion process. The model shows that diffusion is a dynamic process with a dynamic coupling between the behaviour of individual agents and the environment in which they operate.

In this Special Issue, firms’ heterogeneity is at the base of the paper by Stefano Brusoni, Lorenzo Cassi and Simge Tuna “Reinventing the tire: knowledge integration between technical change and strategy making”. In the article, heterogeneity is examined in terms of the different strategies that Pirelli and Michelin (two of the tire industry’s major companies), followed to exploit robotized modular manufacturing, a radical process innovation. Of the two firms, Pirelli, the technological follower, was more successful because it had a more nuanced strategy due to its superior knowledge integration capabilities. By examining the structural characteristics and evolution of inventors’ networks in the two companies, the authors are able to take into account knowledge integration capabilities. Pirelli leveraged a more connected, cohesive and structured skills than Michelin and developed and deployed a more complex strategy that could better fit the characteristics of the new process technology.

One last area in which Luigi has been involved in the last part of his life has been the development of a second generation of evolutionary models: history friendly models. Please see in this respect the book “Innovation and the Evolution of Industries: History Friendly Models” (with F. Malerba, R. Nelson and S. Winter), Cambridge University Press, 2016; and the articles “A History-Friendly Model of the Evolution of the Computer Industry”, (with F. Malerba, R. Nelson and S. Winter), *Industrial and Corporate Change*, 1999. “Innovation and Market Structure in the Dynamics of the Pharmaceutical Industry and Biotechnology: Towards a History-Friendly Model” (with F. Malerba), *Industrial and Corporate Change*, 2002. History friendly models represent a second generation of evolutionary models that focus on the evolution of industries, the dynamics of technologies and industrial change and pay attention to the specificities and histories of industries. In these models a dialogue between empirical analyses of industries, appreciative theorizing derived from these analyses and formal models is developed. The result is that history-friendly models are empirically-grounded, based on a specific empirical evolution of an industry, and aim to capture the causal arguments of the appreciative theory in a stylized and simplified form. The building of the model provides a vehicle for checking out the consistency and relevance completeness of the arguments presented in the appreciative theory. Like most evolutionary models, history-friendly models take the form of computer simulations, and are “agent-based modelling”.

Some of history-friendly models however do not concern industry evolution but examine specific topics in innovation and industrial dynamics. Among these topics, some models analyse the role of demand. A model examines the role of experimental users able to nurture a new technology that may then improve over time and challenge the dominant technology (see “Demand, Innovation and the Dynamics of Market Structure: the Role of Experimental Users and Diverse Preferences” (F. Malerba, R. Nelson L. Orsenigo and S. Winter, *The Journal of Evolutionary Economics,* 2007). Another model looks at the presence of a variety of market segments that allow entrants to compete in environmental niches that are rather separate one from another (see “Technological regimes and demand structure in the evolution of the pharmaceutical industry”, C. Garavaglia, F. Malerba, L. Orsenigo and M. Pezzoni, *The Journal of Evolutionary Economics,* 2012).

Following this line of enquiry on the role of demand through empirically grounded models, in this special issue Herbert Dawid, Gabriele Pellegrino, and Marco Vivarelli present the paper “The role of demand in fostering product vs process innovation: a model and an empirical test”. The major point of the article is that while the innovation literature has provided extensive empirical evidence of the so-called “demand-pull” effect, the impact of demand on product vs process innovation activities has not been examined. The authors develop a formal model that predict that demand has a larger impact in fostering product rather than process innovations. The predictions of the model are tested empirically through a micro-econometric model, which controls for the persistence of R&D and the sectoral specificities. Results are consistent with the model.

Looking at this oeuvre of scientific and scholarly work (nicely reflected on by the papers in this Special Issue), the advancements of our understanding of the dynamics of industries in general, and of biotechnology in particular, so much pushed and led by Luigi are not only impressive but agenda setting. Remarkable above all is Luigi’s ability to putting empirical regularities into a dynamic instead of a static approach in a clever way, developing a sense for, and understanding of the endogenous forces of change, and striping the complexity of real world phenomena down to a set of dynamic mechanisms and forces. That requires a sound understanding of economic theory, a good sense of scientific ingenuity, as well as a superb competence to creatively put together pieces of puzzles. Luigi quite obviously demonstrated such abilities in a superb way and his contributions to advancing the theory and empirics of industrial dynamics and evolution have been outstanding and path-breaking.

So far, this Introduction has been mainly centred on Luigi’s scientific and scholarly work. In the following pages we expand the scope of the tribute to Luigi, by including the talk given by one of us at the opening of the Conference in honour of Luigi Orsenigo in 2018. The Conference gathered his friends, colleagues and former students, and represented a significant testimony of his work, personality and social life.

## Publications of Luigi Orsenigo

- Books
  1. *Innovation and the Evolution of Industries:* *History Friendly Models”* (with F. Malerba, R. Nelson and S. Winter), Cambridge University Press, 2016
  2. *Leveraging Science for Innovation. Swedish Policy for University-Industry Collaboration* (with M. Jacob), 1990–2005, SNS, Stockholm, 2007
  3. *The Economics of Biotechnology* (with M. Mc Kelvey (eds.)), Edward Elgar, Cheltenham, 2006
  4. *Tra continuita’ e cambiamento. La storia dell’Agip Petroli* (with G. Sapelli and P. Toninelli), Bologna, Il Mulino, 1993
  5. *The Emergence of Biotechnology. Institutions and Markets in Industrial Innovation*, Pinter Publishers, London, 1989

b) Papers
  1. History friendly models: retrospective and future perspectives (with G. Capone, F. Malerba, R. Nelson and S. Winter), *Eurasian Business Review *(2019) 9:1–23
  2. Spinoffs in context (with G. Capone and F.Malerba), *Industrial and Corporate Change*, 2019
  3. Industrial Policies for Biotechnology: Limits and New Perspectives, *Journal of Economic Policy*, 2016
  4. The Evolution of the Pharmaceutical Industry” (with F. Malerba)*, Business History,* Special Issue on “Making Sense of Today’s Structures, by Re-introducing Evolutionary (and Institutional) Theory to Business History”, 2015
  5. A Simulation Model of the Evolution of the Pharmaceutical Industry (with C. Garavaglia, F. Malerba and M. Pezzoni), *Journal of Artificial Societies and Social Simulation*, 2013
  6. Are Switching Costs Always Effective in Creating First Mover Advantage? The Moderating Role of Demand and Technological Regimes (with G. Capone and F. Malerba), *Long Range Planning,* 2013
  7. Innovation and market structure in the evolution of the pharmaceutical industry: a history friendly model, (with C. Garavaglia, F. Malerba and M. Pezzoni), *Journal of Economics and Statistics*, 2013
  8. Technological regimes and demand structure in the evolution of the pharmaceutical industry (with C. Garavaglia, F. Malerba and M. Pezzoni), *The Journal of Evolutionary Economics,* 2012
  9. User-producer relations, innovation and the evolution of market structures under alternative contractual regimes (with F. Malerba), *Structural Change and Economic Dynamics*, 2010
  10. In Defence of the Linear Model: An Essay (with M. Balconi and S. Brusoni), *Research Policy*, 2010
  11. Technological Revolutions and the Evolution of Industrial Structures. Assessing the Impact of New Technologies upon Size, Patterns of Growth and Boundaries of Firms, (with G. Dosi, A. Gambardella, M. Grazzi), *Capitalism and Society*, Berkeley Electronic Press, 2008
  12. Beyond market failures: IAVI and the organizational challenges of vaccine development (with S. Brusoni and E. Cacciatori), *Health Partnerships Review*, 2008
  13. Editorial, *International Journal of Biotechnology*, Special Issue on The Evolution of the Life Science Industries (ed. with J. Tait)**,** 2008
  14. Special Issue on The Evolution of the Life Science Industries (ed. with J. Tait), *International Journal of Biotechnology*, 2008
  15. Public Policies and Changing Boundaries of Firms in a “History Friendly” Model of the Co-Evolution of the Computer and Semiconductors Industries (with F. Malerba, R. Nelson and S. Winter), *Journal of Economic Behaviour and Organisation*, 2008
  16. The Italian Connection: the origins of Giovanni Dosi’s thinking and a note on some lost, or never written, manuscripts (with L. Marengo*), Industrial and Corporate Change*, 2008
  17. A History-Friendly Model of the Co-Evolution of the Computer and Semiconductor Industries: Capabilities and Technical Change as Determinants of the Vertical Scope of Firms in Related Industries (with F. Malerba, R. Nelson and S. Winter), *Industrial and Corporate Change*, 2008
  18. A Critical Assessment of Regional Innovation Policy in Pharmaceutical Biotechnology (with A.Rosiello), *European Planning Studies*, 2008, pp. 337–357
  19. Testing Gibrat’s Law: A Bayesian Approach to the Study of Firms’ Growth (with E. Cefis and M. Ciccarelli), *Structural Change and Economic Dynamics,* 2007
  20. Demand, Innovation and the Dynamics of Market Structure: the Role of Experimental Users and Diverse Preferences (with F. Malerba, R. Nelson and S. Winter), *The Journal of Evolutionary Economics,* 2007
  21. The International AIDS Vaccine Initiative (IAVI) in a Changing Landscape of Vaccine Development: A Public – Private Partnership as Knowledge Broker and Integrator (with J. Chataway, S. Brusoni, E. Cacciatori and R. Hanlin), *European Journal of Development Research*, 2007
  22. History Friendly Models of Industrial Evolution: Aims, Applications and Pitfalls, *Revista de Economia*, 2003
  23. Variables Influencing Industrial Funding of Academic Research in Italy: an Empirical Analysis (with G. Bruno), *International Journal of Technology Management*, 2003
  24. The Intensity of Competition After Patent Expiry in Pharmaceuticals. A Cross-Country Analysis (with L. Magazzini and F. Pammolli), *Revue d’Economie Industrielle*, 2002
  25. Innovation and Market Structure in the Dynamics of the Pharmaceutical Industry and Biotechnology: Towards a History-Friendly Model (with F. Malerba), *Industrial and Corporate Change*, 2002
  26. History-Friendly Models: An Overview of the case of the Computer Industry (with F. Malerba, R. Nelson and S. Winter), *Journal of Artificial Societies and Social Simulation*, 2001
  27. The Persistence of Innovative Activities: A Cross-Country and Cross-Sectors Comparative Analysis (with E. Cefis), *Research Policy*, 2001
  28. The (failed?) development of a biotechnology cluster. The case of Lombardy, *Small Business Economics*, 2001
  29. Technological Change and the Dynamics of Networks of Collaborative Relations. The Case of the Bio-pharmaceutical Industry (with F. Pammolli and M. Riccaboni), *Research Policy*, 2001
  30. Mente e società: soluzione di problemi e organizzazioni (with C. Delaini, A. Legrenzi e L. Marengo), *Sistemi Intelligenti*, 2000
  31. Competition and industrial policies in a “history-friendly” model of the evolution of the computer industry (with F. Malerba, R. Nelson and S. Winter), *International Journal of Industrial Organization*, 2000
  32. Knowledge, Innovative Activities and Industry Evolution (with F. Malerba), *Industrial and Corporate Change*, Special Issue on “The Codification of Knowledge” (eds: P. Cohendet and W.E. Steinmueller), 2000
  33. Technological regimes and Schumpeterian patterns of innovation (with S. Breschi and F. Malerba), *The Economic Journal*, April, 2000
  34. A History-Friendly Model of the Evolution of the Computer Industry (with F. Malerba, R. Nelson and S. Winter), *Industrial and Corporate Change*, 1999
  35. The evolution of the forms of organization of innovative activities in biotechnology (with P. Barbanti e A. Gambardella), in *International Journal of Biotechnology*, 1999
  36. Technological Entry, Exit and Suvival (with F. Malerba), *Research Policy*, 1999
  37. The Dynamics of Knowledge and the Evolution of an Industry Network (with Fabio Pammolli, Andrea Bonaccorsi, Massimo Riccaboni and G. Turchetti), *Journal of Management and Governance*, 1998
  38. Technological Persistence and Heterogeneity of Innovative Activities, Sectoral Patterns of Innovation and International Specialization (with F. Malerba and P. Peretto), *The International Journal of Industrial Organization,* 1997
  39. Technological Regimes and Sectoral Patterns of Innovative Activities (with F. Malerba), *Industrial and Corporate Change, Special Issue on Technological Regimes and the Evolution of Industrial Structures*, edited by G. Dosi, F. Malerba and L. Orsenigo, 1997
  40. Industrial Dynamics: Stylized Facts, Empirical Evidence and Theoretical Interpretations (with G. Dosi and F.Malerba), *Industrial and Corporate Change, Special Issue on Technological Regimes and the Evolution of Industrial Structures”*, edited by G. Dosi, F. Malerba and L. Orsenigo, 1997
  41. Special Issue on Technological Regimes, Industrial Demography and the Evolution of Industrial Structures*, Industrial and Corporate Change,* edited by G. Dosi, F. Malerba and L. Orsenigo, 1997
  42. Technological regimes, patterns of innovative activities and industrial dynamics. A survey of empirical evidence and of some theoretical models, *Cahiers de Economie et Sociologie Rurale*, 1996
  43. The Dynamics and Evolution of Industries (with F. Malerba), *Industrial and Corporate Change*, Vol.5, n.1, 1996
  44. Schumpeterian patterns of innovation are technology-specific (with F. Malerba), *Research Policy,* 1996
  45. Choice and action (with D. Lane, F. Malerba and R. Maxfield), *The Journal of Evolutionary Economics*, 1995
  46. Technological Regimes, Selection and Market Structures (with G. Dosi, O. Marsili, R. Salvatore), *Small Business Economics*, 1995
  47. Schumpeterian patterns of innovation (with F. Malerba), *Cambridge Journal of Economics,* 1995
  48. Scientific gatekeepers and industrial development in biotechnology (with N. Buratti and A. Gambardella), *Biotechnology Review*, Special Issue of *The International Journal of Technology Management*, 1993
  49. Technological Regimes and Firm Behaviour”, (with F. Malerba), *Industrial and Corporate Change*, 1993
  50. Regularidades en las actividades de innovacion: una investigacion preliminar para cuatro paises europeos, *Ekonomiaz*, 1992
  51. Competenze tecnologiche nei settori ad alta tecnologia, (with F. Malerba), Centro Studi Confindustria, *CSC Ricerche*, n. 112, Roma, 1995
  52. The evolution of strategy and structure of a state-owned company: the case of Agip Petroli (1960-1990) (with G. Sapelli and P. Toninelli), *Business and Economic History*, 1992
  53. Concorrenza e collaborazione tra imprese nel processo innovativo: il caso della biotecnologia, *L’impresa*, no. 4, 1989
  54. Innovation, Diversity and Diffusion: A Self-Organisation Model (with G. Silverberg and G. Dosi), *The Economic Journal*, Vol. 98, December 1988
  55. Tecnologie emergenti e politica industriale in Italia (with P.Adams), *Rivista Trimestrale di Scienze dell’ Amministrazione,* n.2, 1988
  56. Modelli di diffusione dell’innovazione, *Politica ed Economia”,* Agosto 1987

iii) Chapters in books
  1. Technological Regimes and Demand Structure in the Evolution of the Pharmaceutical Industry (with C. Garavaglia, F. Malerba and M. Pezzoni), in“: Andreas Pyka, Esben Sloth Andersen (eds.) Long Term Economic Development, Demand, Finance, Organization, Policy and Innovation in a Schumpeterian Perspective, Springer, 2013
  2. IPRs, Public Health and the Pharmaceutical Industry. Issues in the Post-2005 TRIPS agenda” (with B. Coriat), in M. Cimoli, G. Dosi, K.E. Maskus, R. L. Okediji and J. H. Reichman, *Intellectual Property Rights: Legal and Economic Challenges for Development*, *Initiative for Policy Dialogue*, Oxford, Oxford University Press, 2014
  3. Pharmaceuticals, in M. Augier and D. Teece (eds.), *The Palgrave Encyclopedia for Strategic Management*, Macmillan, London, 2013
  4. Technological Revolutions and the Evolution of Industrial Structures. Assessing the Impact of New Technologies upon Size, Patterns of Growth and Boundaries of Firms”, (with G. Dosi, A. Gambardella, M. Grazzi), *Capitalism and Society* 3.1 (2008)
  5. IPRs, technological and industrial development and growth: the case of the pharmaceutical industry (with F. Laforgia and F. Montobbio), in N. Netanel (ed.), “*The Development Agenda: Global Intellectual Property and Developing Countries*”, Oxford University Press, 2008
  6. Innovate or Die? A critical review of the literature on innovation and performance (with S. Brusoni and E. Cefis), in Y. Cassis and A. Colli (eds), *Business Performance in the twentieth Century: A Comparative Perspective*, Cambridge University Press, Cambridge, UK, 2008
  7. “History Friendly” Models of Industrial Evolution, in H. Hanusch and A. Pyka (eds.), *The Elgar Companion to Neo-Schumpeterian Economics*, Elgar, Cheltenham, 2007
  8. The International AIDS Vaccine Initiative (IAVI) in a changing landscape of vaccine development: a Public Private Partnership as knowledge broker and integrator (with Chataway, J., Brusoni, S., Cacciatori, E., and Hanlin, R.) in Chataway, J.C., Mackintosh, M. and Wuyts, M. (eds.) *Promoting innovation, productivity and industrial growth and reducing poverty: bridging the policy gap* London: Routledge,. (2008)
  9. Clusters and Clustering in Biotechnology: Stylised Facts, Issues and Theories. From Clusters to network structures and their dynamics, in P. Braunerhjelm and M. Feldman (eds.), *The Genesis of Clusters of Innovation*, Oxford, Oxford University Press, 2006
  10. A history-friendly model of innovation, market structure and regulation in the age of random screening in the pharmaceutical industry (with F. Malerba), in C. Antonelli, D. Foray, B. H. Hall and W.E. Steinmueller (eds.), *New Frontiers in the Economics of Innovation and New Technology. Essays in Honour of Paul A. David*, Edward Elgar, Cheltenham, 2006
  11. The Dynamics of Knowledge Accumulation, Regulation and Appropriability in the Pharma-Biotech Sector: Policy Issues (with G. Dosi and M. Mazzucato), in G. Dosi and M. Mazzuccato (eds.), *Knowledge Accumulation and Industry Evolution. The Case of Pharma-Biotech”*”, Oxford University Press, Cambridge, Cambridge University Press, 2006
  12. Entry, Market Structure and Innovation in a History-Friendly Model of the Evolution of the Pharmaceutical Industry (with C. Garavaglia and F. Malerba), in G. Dosi and M. Mazzuccato (eds.), *Knowledge Accumulation and Industry Evolution. The Case of Pharma-Biotech*”, Oxford University Press, Cambridge, Cambridge University Press, 2006
  13. Biotechnologies et industrie pharmaceutique. Un modéle évolutionaire conforme à l’histoire (with F. Malerba), *Revue del’OFCE*, 2006
  14. Heterogeneity in Firms’Growth in the Pharmaceutical Industry (with E. Cefis and M. Ciccarelli), in G. Dosi and M. Mazzuccato (eds.), *Knowledge Accumulation and Industry Evolution. The Case of Pharma-Biotech*, Cambridge, Cambridge University Press, 2006
  15. Changing Boundaries of firms in the evolution of the computer industry: towards a history-friendly model (with F. Malerba, R. Nelson and S. Winter), in M. McKelvey and M. Holmen (eds.), *Flexibility and Stability in the Evolving Economy*, Oxford University Press, 2005
  16. Prefazione, in F.M. Lombardi and F. Squazzoni (eds.), *Saggi di economia evolutiva*, Franco Angeli, Milano, 2005
  17. Modelli “history-friendly” della evoluzione industriale, *Imprese e Storia*, 31, gennaio-giugno 2005
  18. History Friendly Models of Industrial Evolution: An Overview, in P. De Gijsel and H. Schenk, *“Multidisciplinary Economics*”, Springer, Dordrecht, The Netherlands, 2005
  19. Reflections and Ways Forward (with H. Kettler e M. McKelvey), in M. McKelvey, A. Rickne and J.L. Hellman, *The Economic Dynamics of Modern Biotechnology*, Edward Elgar, London, 2005
  20. Pharmaceuticals Analyzed through the Lens of a Sectoral Innovation System (with M. McKelvey and F. Pammolli), in F. Malerba (ed.), *Sectoral Systems of Innovation*, Cambridge University Press, Cambridge, 2004
  21. Los “modelos amistosos de la istoria” en el analisis de la evolucion de las industria: objetivos y aplicaciones, *Informacion Comercial Espanola*”, 2004
  22. Technology and the Economy (with G. Dosi and M. Sylos Labini), in N. Smelser and R. Swedberg, *Handbook of Economic Sociology*, Cambridge (Mass.), Russell Sage, 2004
  23. Firm Capabilities, Competition and Industrial Policies in a History-Friendly Model of the Computer Industry (with F. Malerba, R. Nelson and S. Winter), in C. Helfat (ed.), *The SMS Blackwell Handbook of Organizational Capabilities. Emergence, Development and Change*, Blackwell, Oxford, 2003
  24. “A behavioral and evolutionary model of the dynamics of the computer industry” (with F. Malerba, R. Nelson and S. Winter), in M. Augier and J. March (eds.), *The Economics of Choice, Change and Organization. Essays in honour of R. Cyert*, Edward Elgar London 2001
  25. Modelli History friendly della evoluzione delle industrie: il caso dell’industria dei computer (with F. Malerba), *Economia e Politica Industriale,* 2001
  26. Product Diversification in a “History-Friendly Model of the Evolution of the Computer Industry (with Franco Malerba, Richard Nelson, and Sidney Winter), in E. Larsen and A. Lomi (eds.), *“Simulating organizational societies.”*, Cambridge (Ma.), MIT Press, 2001
  27. The Determinants of Research Productivity in Pharmaceuticals and Biotechnology: Implications for Public Policies (with I. Cockburn, R. Henderson and G. Pisano), paper prepared for the STEP Board Conference on Technological and Non-technological Factors in Industry Performance, Washington D.C., 19,997; D. Mowery (ed.), *The Competitive Renaissance of the US Industry*, Washington DC, The National Academy, 2000
  28. The Pharmaceutical Industry and the Revolution in Molecular Biology: Exploring the Interactions Between Scientific, Institutional and Organizational Change (with R. Henderson and G. Pisano), in *The Sources of Industrial Leadership*, edited by D. Mowery and R. Nelson, Cambridge, Cambridge University Press, 1999
  29. Organizational Innovation and Organization of Innovative Activities in a Global Economy: the impact on competiveness and growth, in Rubenson, K. and Schuetze H. (eds.)., Transition to the knowledge society, University of British Columbia Press, 1999
  30. Technological Entry and Diversification in Europe, the USA and Japan: 1978–91 (with F. Malerba), in A. Gambardella and F. Malerba F.(ed) *The organisation of innovative activities in Europe,* Cambridge, Cambridge University Press, 1999
  31. Le relazioni università-industria in Italia (con E. Cancogni), in *Conoscenza tecnologica. Nuovi paradigmi dell’innovazione e specificità italiana* (edited by C. Antonelli), Edizioni Fondazione Agnelli, Torino, 1999
  32. Le biotecnologie in Italia: una occasione perduta? (with A. Gambardella), in Bussolati, c., Malerba, F. e Torrisi, S., *Evoluzione industriale e alta tecnologia in Italia: entrata tempestiva, declino e opportunita’ di ricupero*, Il Mulino, Bologna, 1996
  33. Technological innovation and international competitiveness in Italy (with F. Malerba), in J. Molero (eds.), *Technological Innovation, Multinational Corporations and New International Competitiveness,* London, Harwood, 1995.
  34. Tra gerarchie politiche e mercati: il caso delle imprese a partecipazioni statali in Italia (acciaio e petrolio) (with M. Balconi e P. Toninelli), in *Gerarchie, potere, mercato*, edited by M. Magatti, Bologna, Il Mulino, 1995.
  35. Macrodynamics and Microfoundations: an Evolutionary Perspective (with G. Dosi), in O. Grandstand (ed.), *Economics of Technology*, Amsterdam, North Holland, 1994
  36. Innovative learning and institutions in the process of development: on the microfoundations of growth regimes (with F. Chiaromonte and G. Dosi), in R. Thompson (ed.), *Learning and Technological Change*, London, Macmillan, 1993
  37. Evolutionary Regimes and Industrial Dynamics (with G. Dosi and F. Malerba), in L. Magnusson (ed.), *Evolutionary Approaches to Economics*, London, Kluwer, 1993
  38. Archipelago Europe - Islands of Innovation. The case of Italy, in Commission of the European Communities, FAST - MONITOR Project, FOP 243, Prospective Dossier N.1: Science, Technology and Economic Cohesion in the Community, Vol. 20–21, Bruxelles, 1991; published in U. Hilpert and D. Hickie (eds). *Archipelago Europe. The Islands of Innovation in Europe*, London, Routledge, 1993
  39. The dynamics of competition in a science-based technology: the case of biotechnology, in D. Foray and C. Freeman (eds.), *Technology and Competitiveness. The dynamics of created advantages*, London, Pinter Publishers, 1992
  40. L’accumulazione delle capacita’ tecnologiche nell’industria italiana (19869-84) (with F. Malerba), in *“Innovazione tecnologica e servizi alle imprese”,* edited by C. Filippini, F. Angeli, Milano, 1992
  41. La competitivita’ tecnologica internazionale dell’industria italiana negli anni ottanta (with F. Malerba e G. Antonel), in Ministero del Commercio Estero e Istituto Nazionale per il Commercio estero, *Conferenza nazionale sul commercio estero. L’internazionalizzazione dell’impresa,* Atti, Vol. II, Roma, 1992
  42. Microeconomic dynamics and macro regularities: an “evolutionary“ approach to technological and institutional change (with F. Coricelli e G. Dosi), in *Technology and Productivity. The Challenge for Economic Policy*, OECD, Paris, 1991
  43. State Policies on Techno-Industrial Innovation in Weaker Economies. The Case of Biotechnology in Britain and Italy (with W. Faulkner), in U. Hilpert (ed.) *State Policy and Techno- Industrial Innovation*, London, Routledge, 1991
  44. L’ evoluzione dell’industria chimica italiana negli anni ‘80 (with G. Mussati e A. Soru); La piccola e media industria chimica in Italia: i risultati dell’indagine” (with A. Soru); “Le grandi multinazionali chimiche in Italia (with A. Soru), in CeRIC-Federchimica, *Struttura, risultati e problemi dell’industria chimica in Italia*, Milano, SC Sviluppo Chimica, 1991
  45. Teoria evolutiva e innovazione industriale: gli anni ‘80 (with F. Malerba), in *Innovazione tecnologica e teoria economica*, edited by M. Amendola, Bologna, Il Mulino, 1990
  46. Processi di formazione di nuove imprese e dinamica industriale: uno schema di riferimento concettuale (with Marco Vivarelli), in *Alle origini dell’imprenditorialita’*, a cura di G. Mussati, Milano, Etas Kompass, 1990
  47. Technological Regimes and Patterns of Innovation: A Theoretical and Empirical Investigation of the Italian Case (with F. Malerba), in M. Perlman and A. Heertje (eds.), *Evolving Technology and Market Structure*, Ann Arbor, Michigan University Press, 1990
  48. Crescita del terziario per l’industria, processi di esternalizzazione e innovazione, in *Il settore dei servizi nella societa’ e nell’economia italiana. Matrice 1970-1988.*, edited by R. Fiori and F. Stellatelli, Milano, CESDIT, 1989
  49. Processi innovativi nelle piccole e medie imprese, in *Tecnologia: collaborare e’ d’obbligo*, edited by G. Mussati, Confindustria, Comitato Nazionale Piccola Industria, Sipi, Roma, 1989
  50. I pattern di sviluppo della biotecnologia: il quadro di riferimento internazionale nei primi anni ‘80, in *Industria italiana e alte tecnologie” *edited by F. Onida and R. Malaman, Milano, *F. Angeli*, 1989
  51. Coordination and Transformation: An Overview of Structures, Behaviour and Change in Evolutionary Environments (with G.Dosi),in G. Dosi, C. Freeman, R. Nelson, L. Soete and G. Silverberg (eds.), *“Technical Change and Economic Theory.”*, Frances Pinter, London,1988
  52. Industrial Structure and Technical Change (with G. Dosi), in A. Heertje (ed.), *“Technology, Innovation and Finance”*, Oxford, Basil Blackwell, 1988; translations in Italian, French, German and Spanish
  53. La riforma del credito agevolato (with P. Ranci e G. Verga) in *Evoluzione della struttura finanziaria, ruolo degli Istituti di credito a medio e lungo termine e tendenze del credito agevolato*, edited by P. Ranci, Assireme, Milano 1983
  54. Il finanziamento delle piccole e medie imprese, in *Evoluzione della struttura finanziaria, ruolo degli Istituti di credito a medio e lungo termine e tendenze del credito agevolato*, edited by P. Ranci, Assireme, Milano 1983
  55. Il finanziamento delle imprese nella programmazione dei flussi finanziari, in *La programmazione dei flussi finanziari*, edited by G. Vaciago, Il Mulino, Bologna, 1983

## Data Availability

There are no data used.

